# Personalized Frequency Modulated Transcranial Electrical Stimulation for Associative Memory Enhancement

**DOI:** 10.3390/brainsci12040472

**Published:** 2022-04-02

**Authors:** Jovana Bjekić, Marko Živanović, Dunja Paunović, Katarina Vulić, Uroš Konstantinović, Saša R. Filipović

**Affiliations:** 1 Human Neuroscience Group, Institute for Medical Research, University of Belgrade, Dr Subotica 4, 11000 Belgrade, Serbia; dunja.paunovic@imi.bg.ac.rs (D.P.); katarina.vulic@imi.bg.ac.rs (K.V.); uros.konstantinovic@imi.bg.ac.rs (U.K.); sasa.filipovic@imi.bg.ac.rs (S.R.F.); 2 Institute of Psychology and Laboratory for Research of Individual Differences, Department of Psychology, Faculty of Philosophy, University of Belgrade, 11000 Belgrade, Serbia; marko.zivanovic@f.bg.ac.rs

**Keywords:** associative memory, transcranial direct current stimulation (tDCS), transcranial alternating current stimulation (tACS), transcranial oscillatory current stimulation (otDCS), personalized brain stimulation, individual theta-band frequency, EEG, cognitive assessment

## Abstract

Associative memory (AM) is the ability to remember the relationship between previously unrelated items. AM is significantly affected by normal aging and neurodegenerative conditions, thus there is a growing interest in applying non-invasive brain stimulation (NIBS) techniques for AM enhancement. A growing body of studies identifies posterior parietal cortex (PPC) as the most promising cortical target for both transcranial magnetic stimulation (TMS) and transcranial electrical stimulation (tES) to modulate a cortico-hippocampal network that underlines AM. In that sense, theta frequency oscillatory tES protocols, targeted towards the hallmark oscillatory activity within the cortico-hippocampal network, are increasingly coming to prominence. To increase precision and effectiveness, the need for EEG guided individualization of the tES protocols is proposed. Here, we present the study protocol in which two types of personalized oscillatory tES–transcranial alternating current stimulation (tACS) and oscillatory transcranial direct current stimulation (otDCS), both frequency-modulated to the individual theta-band frequency (ITF), are compared to the non-oscillatory transcranial direct current stimulation (tDCS) and to the sham stimulation. The study has cross-over design with four tES conditions (tACS, otDCS, tDCS, sham), and the comprehensive set of neurophysiological (resting state EEG and AM-evoked EEG) and behavioral outcomes, including AM tasks (short-term associative memory, face–word, face–object, object-location), as well as measures of other cognitive functions (cognitive control, verbal fluency, and working memory).

## 1. Introduction

Associative memory (AM) is the ability to remember multiple units of information bound together [1]. The process of binding, that is central to AM [2,3], enables remembering of the relationships between two or more items or between an item and its context. This allows one to create complex memories of people, places, and events, as well as acquire new skills and knowledge that we rely on when making everyday judgments and decisions [4]. Unfortunately, AM is affected by different neurological states and conditions—it declines with normal ageing [5], and its impairment has been identified as one of the early and prominent symptoms of mild cognitive impairment (MCI) and dementia [6,7]. The AM tests have been even proposed as assessment and diagnostic tools for MCI and Alzheimer’s disease [8,9]. Due to generally weak response of memory deficits to pharmacological treatment [10,11], interest in development and application of different non-invasive brain stimulation (NIBS) techniques and protocols to promote AM, ultimately aiming at NIBS treatments for memory deficits [12,13,14], has sparked. For broader review of NIBS techniques and their application across different neurological states and conditions see [15,16,17,18].

Hippocampus with the surrounding medial temporal structures is the central hub for AM [3,19,20,21], but the formation and retention of memory representations are achieved through interconnectivity of a widespread cortico-hippocampal network, including frontal, temporal, and parietal cortices [22]. Initial transcranial electrical stimulation (tES) studies, which focused on AM, applied constant anodal current over the frontal lobe, primarily targeting dorsolateral prefrontal cortex (for reviews, see Galli et al. [23], Kim et al. [24], and Manenti et al. [25]). However, these studies resulted in mixed findings and questionable specificity of the effects [23,24,26]. In their seminal study, Wang and colleagues showed that MRI-guided transcranial magnetic stimulation (TMS) of posterior parietal cortex (PPC) led to increased hippocampal activation and strengthening of parieto-hippocampal functional connections, which resulted in AM enhancement [27]. The functional relevance of PPC-hippocampus relay for AM enhancement was further established in series of follow-up TMS experiments [28,29,30,31,32,33], which complemented previous imaging and neuropsychological evidence [34,35,36,37,38]. Building up on that, tES studies showed that anodal stimulation of PPC leads to better AM of faces and words [39,40], as well as AM of objects and locations [39,41]. However, some studies did not find the modulatory effects of PPC-targeted transcranial direct current stimulation (tDCS) for AM-like functions [42,43,44].

In the attempt to target AM specifically, one could aim to induce entrainment of the endogenous neural oscillations, using different oscillatory tES protocols such as transcranial alternating current stimulation (tACS) and transcranial oscillatory current stimulation (otDCS). Namely, unlike the standard anodal tDCS with constant current flow between two opposing electrodes [45], tACS applies an oscillating current that switches polarity [46]. As a result, tDCS has been shown to increase cortical excitability by modulation of resting state membrane potential [16], while the rhythmic changes of the current intensity and polarity in tACS are assumed to entrain neural oscillations to the stimulation frequency by alternating membrane potentials between depolarization and hyperpolarization [47,48]. The combination of these two modes of action led to the development of otDCS, in which the current oscillates at a certain frequency around the set value, but always stays in the same polarity [49]. Therefore, anodal otDCS should provide entrainment-like effects in addition to the change in the excitability, and consequently enhance cognitive functions [50].

Modulatory effects of tACS have been shown for a wide range of cognitive functions including attention, executive functions, working memory, etc. (see Klink et al. [51], Booth et al. [52] for recent reviews). In contrast, the otDCS is a relatively novel approach that has been used in just a handful of studies so far. Building up on the evidence of functional link between theta-band oscillations (4–8 Hz) and hippocampal binding in AM [4], we found positive effects on AM following 20 min of 5 Hz otDCS [50], while Lang and colleagues showed increased AM performance when high definition 6 Hz tACS was applied during the encoding [53]. Finally, Meng and colleagues showed that 6 Hz tACS over the PPC can selectively affect associative, but not item encoding [54]. Despite the promising initial findings, the mechanisms behind oscillatory tES induced changes and behavioral effects on AM are yet to be systematically assessed.

Another important challenge is the interindividual variability in responsiveness to tES. There has been ample evidence that people respond differently to stimulation [55,56,57], which translates to unreliable cognitive outcomes, often seen in the literature. Age, brain development, functional, and anatomical features of the brain and head all may influence the density and distribution of electrical currents applied by tDCS and the effects of stimulation, as a result [58]. Moreover, the level of individual cortical excitability as well as the individual structural and default functional organization of the brain may contribute to variable results [56,58]. To overcome these issues, the need for the development of personalized tES protocols has emerged [59,60,61]. One possible direction in tES personalization is to use electroencephalography (EEG) to extract information about the relevant neurophysiological properties of one’s brain, and then to use it to build individually adjusted tES protocol [62]. For oscillatory tES protocols, this usually means setting the frequency of the oscillations to one’s individual alpha frequency (IAF) [63,64], individual gamma-band frequency (IGF) [65], individual beta-band frequency [66] or, in the case of memory, the individual theta frequency (ITF). The tACS at ITF has been applied to modulate working memory [67], intelligence [68], and cognitive control [69]. However, the ITF-based tES protocols have not been used for AM modulation so far.

Therefore, we set up to develop personalized tES protocols for targeted AM modulation and to comprehensively assess their neurophysiological and behavioral effects. Specifically, we designed the experiment in which we would use EEG data acquired during AM tasks to extract ITF, which would then be used to personalize tACS and otDCS. To comparatively assess the effects of different tES protocols, we apply constant anodal tDCS, ITF-tACS, ITF-otDCS and sham tDCS to each participant and measure resting state and task-evoked EEG together with behavioral performance on several AM tasks, as well as tasks tapping other cognitive functions.

## 2. Materials and Methods

**Figure 1:**
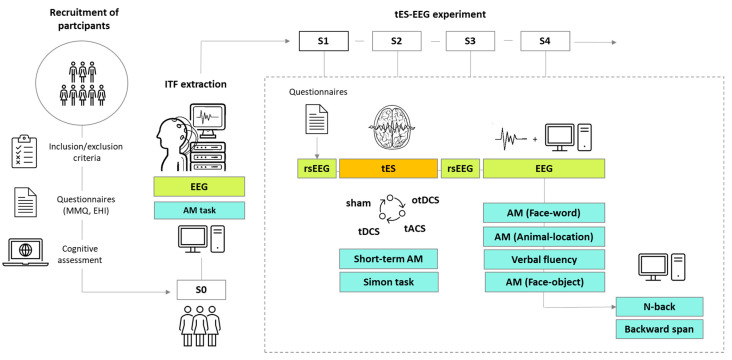
The overview of the study design—recruitment and the selection of participants; first experimental session (S0) in which ITF is determined based on EEG data recorded during the AM task; tES-EEG experiment consisting of four experimental sessions (S1–S4) for each participant (counterbalanced cross-over design). Each of S1–S4 adopts the same experimental procedure: (1) questionnaires, (2) resting state EEG (rsEEG), (3) one of tES protocols (sham/tDCS/tACS/otDCS), during which participants performed Short-term AM task first and the Simon task second, (4) rsEEG, (5) Face–word AM task with EEG recording, (6) Animal–location AM task with EEG recording, (7) verbal fluency task, (8) Face–object AM task with EEG recording, (9) n-back task, and (10) Backward span task.

The study has 3 phases—selection and recruitment of participants (see Section 2.1), followed by the initial AM assessment and ITF extraction (see Section 2.2), and, finally, the within-subject cross-over combined tES-EEG experiment (see Section 2.3). In other words, the study consists of web-based screening with cognitive pre-assessment, and five experimental sessions conducted in the lab (Figure 1)—one EEG recording session only (S0) and four sessions in which distinct types of tES are applied (S1–S4). The study is designed in line with the guidelines of the Declaration of Helsinki and is approved by the Institutional Ethics Board (EO129/2020). Written informed consent is obtained from all participants and can be revoked at any time during the study.

### 2.1. Selection and Recruitment of Participants

The sampling population is young healthy adults of both genders, who satisfy the inclusion criteria and are willing to take part in the study in return for monetary compensation (8 €/h). To determine an adequate sample size for the study, *a priori* power analysis in G*Power [70] is performed. In line with analytical strategy outlined in Section 2.5., planned contrasts (2 levels) with *r* = 0.50 between repeated measures (see Section 2.4.9) will have the power of 0.95 at alpha error probably of 0.05 to detect η_p_^2^ = 0.15 with 21 participants. This is similar to the power of previous studies assessing tES effects on AM [39,40,50], which reported effects in the range of 0.18–0.30. Since there are no previous studies that could directly inform on the expected effects sizes for different outcome measures, we conducted additional power analysis to determine the minimal sample size needed to detect medium effects (i.e., Cohen d = 0.50/η_p_^2^ = 0.06) [71]. The same statistical analysis (2-levels, planned contrasts) will have the power of 0.80 to detect medium effect size of η_p_^2^ = 0.06 at an alpha error probably of 0.05 with 33 participants.

The recruitment is conducted through an open call for participation through different University platforms (laboratory mailing lists, social media platforms, and student organizations). After expressing an interest, all potential participants are sent the online eligibility self-assessment checklist. The inclusion criteria for the study are that participants are right-handed, with normal or corrected-to normal vision, between 20 and 35 years of age. The exclusion criteria include history of seizures, neurological or psychiatric disorders, cognitive or learning disability, traumatic head and brain injury, metal implants in the head, chronic or acute skin condition, pregnancy, and use of psychoactive substances and medication. Those who satisfy the inclusion criteria are invited to complete a battery of cognitive tests and additional questionnaires.

The pre-experiment cognitive assessment includes short measures of fluid intelligence (*Gf*), crystalized intelligence (*Gc*), visual processing (*Gv*), and processing speed (*Gs*) as defined within Cattell–Horn–Carroll’s model of intelligence [72]. For the assessment of *Gf*, *Gc*, *Gv* and *Gs*, we use short Raven’s matrices [73], Synonym test [74], Spatial abilities test [74], and Identical figures test [74], respectively [75]. In addition to cognitive assessment, the potential participants are administered Multifactorial Memory Questionnaire (MMQ) [76], with three scales measuring satisfaction with memory functioning (18 items), self-appraisal of memory ability (20 items), and self-reported use of external and internal memory strategies (19 items) [77]. Finally, participants are asked to complete Edinburgh Handedness Inventory [78] and report if they had previous experience with EEG and/or different NIBS techniques.

Out of 51 participants who expressed interest to take part in the study, one did not satisfy the inclusion criteria, five did not complete pre-experiment cognitive assessment, and three did not come to the initial experimental session (S0)—thus the sample of 42 participants (26 females), age 22–34 years (*M* = 25.05, *SD* = 3.55), is enrolled in the study.

### 2.2. Experimental Set-Up and Equipment

The light, mobile, battery-operated, hybrid tDCS-EEG Starstim device (Neuroelectrics Inc., Barcelona, Spain) is used for both EEG acquisition and tES. The Starstim device is attached to the back of the neoprene cap and remotely operated via Neuroelectrics^®^ Instrument Controller (NIC2) software (Neuroelectrics Inc., Barcelona, Spain) that enables real-time monitoring of EEG signal as well as creating and delivering customized tES protocols. For computerized cognitive assessment, the tasks are programmed and administered in OpenSesame software [79], version 3.2.8. The Transmission Control Protocol (TCP) is used to maintain connection and synchronization between NIC and OpenSesame. The OpenSesame code for TCP enabled EEG triggers (stimulus onset and participants’ responses) is available in the Supplementary Material.

### 2.3. S0: EEG Recording for Initial AM Assessment and ITF Extraction

The first experimental session (S0) is comprised of EEG recording, during which the initial AM assessment is conducted, with the primary objective to extract ITF that will be used for tACS/otDCS frequency-personalization in the subsequent sessions.

The EEG is recorded with Ag/AgCl electrodes (4 mm diameter, 1 cm^2^ gel-contact area) from 20 positions (Fp1, Fp2, Fz, F3, F4, F7, F8, T7, T8, Cz, C3, C4, CP5, CP6, Pz, P3, P4, PO7, PO8 and Oz, according to the International 10-10 EEG positioning system). The EEG signals are recorded with the sampling rate of 500 Hz, 0–125 Hz (DC coupled) bandwidth, and 24 bits—0.05 µV resolution. First, 10 min of resting state EEG is recorded (5 min eyes closed and 5 min eyes open), followed by the AM assessment.

The AM task consists of encoding and recognition block. Similar to the Lang et al. [53], pictures of faces and landscapes are used as stimuli. In the encoding block, 42 face–landscape pairs are presented in successive manner for 2000 ms with the varying inter-stimulus interval (ISI) (i.e., white screen with fixation point lasting between 1250–1750 ms). For the recognition block, 42 face–landscape pairs from the encoding block are intermixed with 42 new pairs, which have been created from the same set of faces and landscapes but rearranged into new pairs (“recombined”). The 84 stimuli are successively presented in a fixed, prerandomized order, and participants’ task is to recognize the pair as either “old” or “recombined”. The AM recognition success rate (% of correctly identified “old” pairs + % of correctly identified “recombined” pairs) is used for the initial AM assessment.

To extract ITF, the EEG recorded during the encoding block of the AM task is analyzed. The offline preprocessing is performed in EEGLAB for MATLAB [80], where the signal is high-pass filtered (0.1 Hz) as well as cleaned from the power-line noise (50 Hz) and eye-movement artefacts. Next, the signal is epoched (−1000 ms to 2500 ms related to the onset of the AM encoding task stimuli) and labeled based on subsequent associative recognition. Only the epochs recorded during presentations of subsequently correctly recognized pairs (i.e., correctly identified “old” pairs) are further processed.

The selected epochs are baseline-corrected to the mean of the pre-stimulus period, and complex Morlet wavelet (7 cycles) transformation with frequencies from 1 to 15 Hz in 0.5 Hz steps is performed. To determine ITF, the event-related spectral perturbation (ERSP) from centroparietal electrodes (Cz, C3, C4, Pz, P3 and P4) is calculated [81], and further expressed as a ratio against the baseline (−800 to −100 ms). Subsequently, for each electrode, the frequency with the highest ERSP value is extracted from 19 overlapping sliding time windows (100 ms width in 50 ms steps) covering the 250 ms to 1250 ms post-stimulus-onset segment. Finally, to find the dominant theta frequency, the modal value (i.e., the most frequently occurring value) of frequencies between 4–8 Hz (in 0.5 Hz steps) in the time x electrode matrix for each participant (114 cells per participant) is calculated. The rationale behind this ITF extraction method and EEG processing pipeline is presented in detail elsewhere [82].

### 2.4. S1–S4: The Assessment of Neurophysiological and Behavioral Effects of tES

To assess the behavioral and neurophysiological effects of different tES protocols, the within-subject cross-over design is adopted (Figure 1). Each type of tES is delivered on separate days in a counterbalanced order (see Section 2.4.1), with at least 7 days between each session to avoid potential carryover effects [83]. The two-experimenter procedure is implemented with one person delivering stimulation and monitoring EEG signal, and the other administering cognitive tasks and questionnaires. However, this is a single blind experiment, since, for technical reasons, one if not both experimenters need to be aware of the type of stimulation to be applied in each session (e.g., in case of any equipment malfunction or disruption).

#### 2.4.1. Counterbalancing

Since each participant is bound to receive four types of stimulation (tACS/otDCS/tDCS/Sham) and complete assessment using parallel forms of cognitive tasks (F1/F2/F3/F4) in separate sessions (S1/S2/S3/S4), we implement Latin square counterbalancing. This means that the participants are split into groups with respect to stimulation order (Group 1: Sham-tDCS-otDCS-tACS, Group 2: tDCS-Sham-tACS-otDCS, Group 3: otDCS- tACS-Sham-tDCS, and Group 4: tACS-otDCS-tDCS-sham), as well as with respect to the task form (Group A: F3-F4-F1-F2, Group B: F4-F3-F2-F1, Group C: F1-F2-F3-F4, and Group D: F2-F1-F4-F3). Therefore, each participant can be assigned the stimulation type and task form for each session (e.g., Participant 1–1A: S1–sham/F3, S2–tDCS/F4, S3–otDCS/F1, S4–tACS/F2; Participant 2–4B: S1–tACS/F4, S2–otDCS/F3, S3–tDCS/F2, S4–sham/F1, etc.). To balance the groups, a quasi-random allocation is performed matching the participants on age, gender, cognitive abilities, and initial AM assessment conducted within S0.

#### 2.4.2. Procedure

Each session (S1–S4) follows the same procedure which lasts approximately 2 h. Upon arrival, the participants fill in the questionnaires (see Section 2.4.3). Next, the Starstim device is attached to the back of the neoprene cap and the stimulation and EEG-recording electrodes are placed (see Section 2.4.5). After the TCP connection is established, brief signal quality and impedance checks are performed. The participant is then left alone in the dimly lit room and instructed to relax and follow the experimenter’s instructions that they are about to receive.

First, participants are instructed to close their eyes for the resting state EEG recording (see Section 2.4.5 and Section 2.4.6). The experimenter monitors the EEG signal and starts the protocol in NIC when typical rhythms of the rsEEG are clearly visible. The NIC protocol consists of 5 min of EEG recoding, followed by 20 min tES period, after which 5 min of EEG is again recorded. During the stimulation, participants are prompted to report the level of stimulation-induced discomfort on a 10-point numerical scale (1—absence of discomfort, 10—extreme discomfort; approximately at minutes 1, 8, 16 and 20). In addition to that, during tES, participants complete two computerized cognitive tasks (see Section 2.4.7 and Section 2.4.8)—a short-term AM task (approximately minutes 3 to 8), and the Simon task afterwards (approximately minutes 11 to 16). The tasks are always given in the same order and are meant to keep participants engaged during most of the tES period. After the EEG-tES-EEG protocol is finished, the participants are asked to fill in the questionnaire with potential stimulation-related side effects (see Section 2.4.3).

Next, the participants go on to complete three AM tasks while EEG is recorded—specifically, the Face–word AM task, the Animal–location AM task, and the Face–object AM task (see Section 2.4.7). Between the Animal–location and Face–object AM tasks, participants are administered short control tasks assessing verbal fluency (see Section 2.4.8). After the AM tasks are completed, the participants are asked to complete two additional tasks, both tapping working memory, during which the EEG is not recorded—namely, the computer administered n-back task and experimenter-administered Backward-span task (see Section 2.4.8). In addition to the instructions embedded in all computerized tasks, before each task, the experimenter provides a brief verbal explanation of the instructions to assure participants’ understanding. Except for the Backward span, all other cognitive tasks are designed to be performed without an experimenter present in the room.

#### 2.4.3. Questionnaires

To monitor for potential confounding effects of mood and current state, at the beginning of each session, participants are asked to complete: (1) the 21-item version of Depression Anxiety Stress Scale (DASS) [84], which is a self-report measure of mood-related symptoms during the past week, and a (2) short questionnaire which includes questions about current mood (scale: −5 [very bad mood] to + 5 [very good mood]), level of tiredness (scale −5 [extremely tired ] to +5 [not tired at all]), hours of sleep during previous night, coffee consumption in last 12 h (number of cups), nicotine consumption in last 4 h (number of cigarettes), and alcohol consumption in the last 24 h (number of drinks). These types of data are collected in line with guidelines for comprehensive reporting in tES experiments [85].

We also include the side-effects self-report questionnaire before and after each tES protocol. This questionnaire asks participants to rate each of the potential side effects on a 10-point scale (1—not at all, 10—extremely): headache, neck pain, back pain, blurred vision, skin irritation, prickling/tingling sensation, itching, increased heart rate, burning sensation, dizziness, acute mood swings, tiredness, and anxiety [83].

#### 2.4.4. Stimulation Protocols: tACS, otDCS, tDCS and Sham

**Figure 2:**
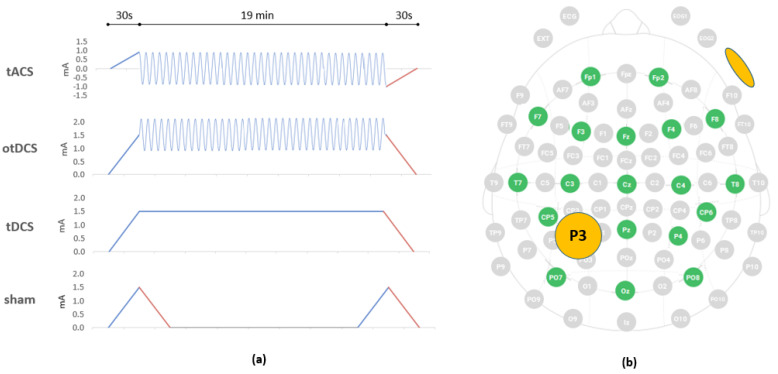
(**a**) The tES stimulation protocols–tACS (30 s gradual ramp up to 1 mA, 19 min of 0 ± 1 mA at ITF, 30 s gradual ramp down), otDCS (30 s gradual ramp up to 1.5 mA, 19 min of 1.5 ± 0.5 mA at ITF, 30 s gradual ramp down), tDCS (30 s gradual ramp up to 1.5 mA, 19 min of 1.5 mA, 30 s gradual ramp down), and sham (30 s gradual ramp up to 1.5 mA, 30 s gradual ramp down to 0 mA, 19 min 0 mA, 30 s gradual ramp up to 1.5 mA, 30 s gradual ramp down); (**b**) the electrode montage—the positions of EEG recording electrodes (green) and the position of tES electrode over left PPC (orange); the return electrode is positioned on the right cheek (orange).

There are four stimulation conditions: tACS, otDCS, tDCS and sham (Figure 2a). The tACS is delivered as 2 mA peak-to-peak (i.e., between −1 mA and +1 mA) sinusoidal oscillating current at ITF (4–8 Hz). The otDCS is delivered in positive polarity (i.e., anodal current) with the current oscillating ±0.5 mA in sinusoidal mode around +1.5 mA (i.e., between +1 mA and +2 mA), also at ITF (4–8 Hz). Anodal tDCS has a constant current intensity of +1.5 mA. All active stimulation protocols have a gradual ramp up/down of 30 s at the beginning and at the end of stimulation period. Finally, in a sham condition, the current is briefly delivered for 60 s at the beginning and at the end of stimulation period (double ramp up/down sham protocol), both with a gradual ramp up to 1.5 mA (30 s) and immediate gradual ramp down (30 s). In each stimulation condition, the protocol is delivered using NIC and lasts 20 min in total.

#### 2.4.5. EEG-tES Electrode Montage

Since we use a hybrid Starstim device (Neuroelectrics Inc., Barcelona, Spain) for both EEG acquisition and tES, the electrodes are placed in the neoprene cap positioning grid with 39 predefined positions based on a subset of the international 10–10 EEG system (Figure 2b). To assure adequate electrode placement, the standard head-measurement is performed to select the fitting size of the neoprene cap (S/M/L) and to mark head locations corresponding to the Cz and P3. Round rubber electrodes (25 cm^2^) inserted in saline-soaked sponge pockets are used for stimulation, while EEG is recorded with gel Ag/AgCl electrodes (4 mm diameter, 1 cm^2^ gel-contact area). To target the left PPC, one stimulation electrode is placed at P3, while the return electrode is secured with medical adhesive tape on the contralateral cheek. The EEG electrodes are placed on Fp1, Fp2, Fz, F3, F4, F7, F8, T7, T8, Cz, C3, C4, CP5, CP6, Pz, P4, PO7, PO8 and Oz. The 19-electrode set-up is selected to avoid contact with stimulating electrode while maintaining the reasonable whole head-coverage. Depending on the quality of the signal, either the dual CMS-DRL ear clip electrode attached to the right earlobe, or two pre-gelled sticktrodes—the CMS on the right mastoid, and the DRL just below—are used.

#### 2.4.6. EEG Acquisition Protocols

In each session, the EEG is recorded at rest and during task performance. The resting state EEG is recorded for 5 min immediately before and 5 min immediately after the stimulation, each time starting with 3 min of eyes-closed and 2 min eyes open. The task-EEG is recorded during each of the AM tasks (Face–word, Animal–location, and Face–object). The stimulus onsets and all keyboard responses are pre-labeled and are automatically recorded as separate events in an EEG file. During the experiment, the signal is continuously monitored via NIC live view. The same as in the S0 session, the EEG signals are recorded with the sampling rate of 500 Hz, 0–125 Hz (DC coupled) bandwidth, and 24 bits–0.05 µV resolution.

#### 2.4.7. Associative Memory (AM) Tasks

**Figure 3:**
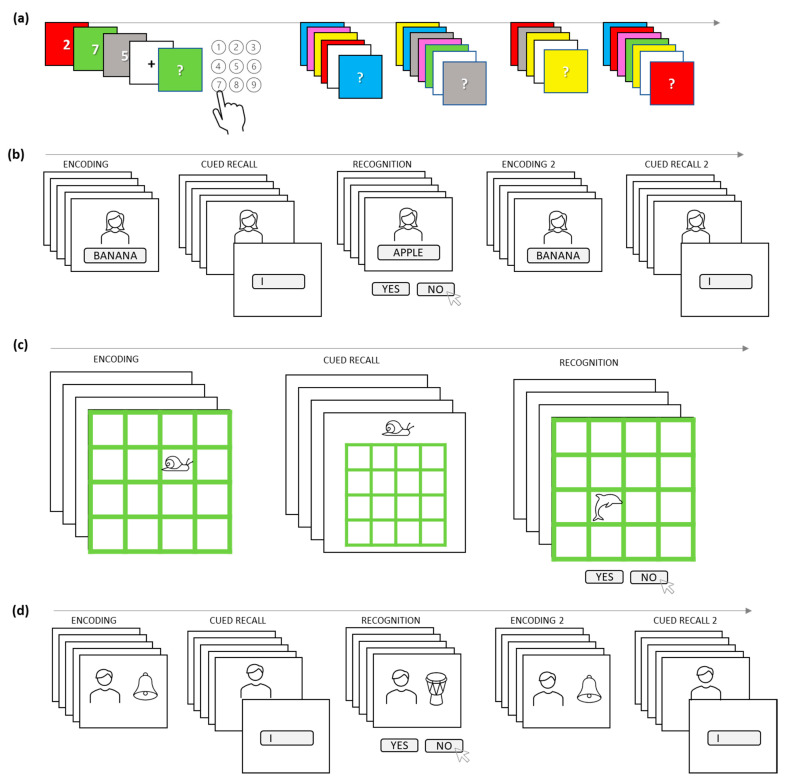
Schemes of the AM tasks employed in the study (**a**) the short-term AM task; (**b**) the Face–word AM task; (**c**) the Animal–location task; (**d**) the Face–object AM task.

To assess the effects of tES, both during the stimulation (so called “online protocol”) as well as immediately after (so called “offline protocol”), we use several AM tasks (Figure 3).

The short-term AM task, which is completed during tES, is a newly developed associative version of the classical Sternberg task [86]. Namely, the stimuli are single-digit numbers (0–9) presented on a differently colored background (green, red, blue, yellow, gray, pink). The color–number trials are sequentially presented for 1250 ms in varying sequence lengths (3, 4 or 5 trials). After each sequence, the participants are shown the color, and they are asked to recall the number associated with a given color from that sequence (Figure 3a). The task has 42 sequences in total and measures the ability to continuously create new associations in short-term memory.

For the offline AM assessment, we use classical paired-associate paradigms tapping the ability to bind novel items, and subsequently recognize correct associations or recall information when cued by one of the stimuli from the pair. Three AM tasks of a similar structure but with different stimuli are designed to potentially tap into shared as well as different cognitive processes.

The Face–word AM task (Figure 3b) consists of five blocks in the following sequence: Encoding–Cued-recall–Recognition–Encoding–Cued-recall. The stimuli are portrait pictures of people from Chicago Face Database [87], and frequently used, easily imaginable words, 4–7 letters long with 2–3 syllables. In the Encoding block, 30 face–word pairs are successively presented for 2000 ms with variable ISI of 1250–1750 ms (fixation point screen). In the Cued-recall block, 30 previously learned faces are presented one-by-one and the participants’ task is to recall and type-in the word which was paired with the presented face in the encoding block. For the Recognition block, the face–word pairs from the encoding block (“old” pairs) are intermixed with new pairs made from rearranging the same words and faces (“recombined” pairs). The participants are successively presented with 60 pairs (30 “old” and 30 “recombined”), with the task to recognize the pair as either “old” or “recombined” and to answer using designated keyboard keys. After the Recognition block, participants are given another opportunity to learn the face–word pairs in the second Encoding block, which is again followed by a Cued-recall block. Both Encoding blocks as well as Cued-recall blocks are identical.

The Animal–location task is a version of an object–location paradigm and is comprised of only three blocks: Encoding–Cued-recall–Recognition (Figure 3c). During the Encoding block, 32 pictures of animals sequentially appear for 2000 ms on one of the 16 positions in a 4 × 4 grid. The participant’s task is to remember the position in which each animal has been shown. In the Cued-recall block, participants are to click (using the mouse) on the grid-position where they think the probe animal appeared in the Encoding block. In the Recognition block, each animal is presented once on the position from the encoding block (“old”) and another time on the new one (“recombined”), thus resulting in 64 trials which one needs to classify as either “old” or “recombined”.

The Face–object AM task follows the same structure as the Face–word AM task: Encoding–Cued-recall–Recognition–Encoding–Cued-recall (Figure 3d). The stimuli are portrait pictures from FACES database [88] and images of the everyday objects from Google images. In the Encoding block, 30 face–object pairs are successively presented (2000 ms, ISI 1250–1750 ms), and the participant’s task is to learn them. In the Cued-recall block, participants are presented with the faces, but this time they are asked to name the object that was presented alongside each face. The Recognition block consists of 60 pairs (30 “old” and 30 “recombined”) and is equivalent to the one in the Face–word AM task. The Encoding and Cued-recall blocks are repeated in the Face–object AM task as well.

#### 2.4.8. Other Cognitive Tasks

To assess the specificity of the potential tES effects, the study design includes additional cognitive tasks. Namely, during tES, the participants complete a version of the Simon task [89], in which the word *left,* or *right* is presented on either the left or right side of the screen, and participants’ tasks are to press the left or right key depending on the meaning of the displayed word, while ignoring its position on the screen. In total, the task has 150 trials—75 congruent (word *left* presented on the left, word *right* presented on the right) and 75 incongruent (word *left* presented on the right, and vice versa). The task is usually used to measure cognitive or motor inhibition, but it can be used as the attention measure too.

As a control-function task for the tES after-effects, we use Verbal fluency, in which participants are given a letter (e.g., R) and their task is to write down as many words as possible starting with a given letter, without repetition, within 90 s. We successfully used this type of task as a control in our previous tDCS-AM experiments [39,40,50].

To assess the focality of the potential effects on memory, two tasks probing working memory are added. The first is a n-back task–specifically the Letter 3-back task [90], in which participants are successively presented with different letters (e.g., B, S, T), and they are instructed to respond via keypress when presented with the letter, which is the same as the one shown three trials before. This type of task is the most prominent behavioral measure of WM in tDCS literature [91]. Finally, we use Backward span as an additional WM measure. In this task, participants listen to a recorded sequence of numbers (e.g., 3-5-8) via earphones, and they are instructed to orally repeat the sequence in reverse order (8-5-3). The length of the sequence increases by one number after every two trials with the stopping rule of two consecutive errors at the same level. This type of WM task is adopted in widely used broad cognitive batteries such as Wechsler Adult Intelligence Scales (WAIS) [92], and thus frequently used in clinical settings.

#### 2.4.9. Parallel Forms of the Tasks

Each of these tasks is developed in four parallel forms (F1/F2/F3/F4). Parallel forms of short-term AM tasks are created by recombining the number–color associations and the presentation sequence. That is, one form of the task serves as a pattern, which was multiplied by substitution (e.g., F1: yellow-3, green-9, gray-4; F2: green-4, gray-1, red-5; F3: gray-5, red-2, blue-6; F4: red-6, blue-3, yellow-7). A similar strategy is adopted for the Simon task, as well as for 3-back and Backward-span tasks. For the verbal fluency assessment, we use different target letters across forms (R/S/P/M).

Creating the parallel forms for remaining AM tasks is a bit more complex. Namely, for the Face–word AM task, 120 faces from the Chicago Face Database [87] are selected and randomly paired with 120 simple words; then, this process is repeated for a Face–object task, this time using the 120 pictures from the FACES database [88] and 120 images of the common objects. It needs to be assured that there were no repetitions between the two tasks—that is, no overlap between the words (Face–word AM task) and typical words that would be used to name the objects (Face–object AM task). Three researchers inspected the pairs and reordered those that formed stereotypical associations. Next, the faces stimuli are split into groups based on the age and gender of the people in pictures, and each group is used to create one form of the task (Face–word AM task–F1: young women, F2: young men, F3: older women, F4: older men; Face–object AM task–F1: older men, F2: older women, F3: young men, F4: young women). This is done to ensure no interference between the two tasks that are completed in the same session, as well as to minimize the interference between different forms of the same task across sessions, and to increase the task difficulty (i.e., to homogenize the stimuli within the task) to minimize the chance of the ceiling effects. To create parallel forms of the Animal–location task, a database of 128 pictures of the animals is created. The animals are grouped into 32 sets of four to be distributed across the task forms (e.g., 4 distinctive pictures of domestic cats, 4 pictures of wild cats, 4 pictures of lizards, 4 pictures of small insects, etc.). Across task-forms, we use different pastel colors for the background of the grid to minimize the interference, and the animal position of the grid as well as the location sequence is multiplied by substitution. In this way, the basic properties of the task are kept the same and each form is distinctive enough to minimize the between-forms transfer or interference.

To ensure that the task forms are parallel, we conducted a pilot study on a convenience sample (*N* = 84, age *M* = 22.13, *SD* = 4.16, 71 female). The pilot study showed only marginal differences between forms in the Face–word AM task [cued recall: *F*_(3,69)_ = 1.35, *p* = 0.265, η_p_^2^ = 0.06, recognition: *F*_(3,69)_ = 2.56, *p* = 0.062, η_p_^2^ = 0.10], minimal differences between forms of Face–object AM task [cued recall: *F*_(3,78)_ = 4.02, *p* = 0.010, η_p_^2^ = 0.13, recognition: *F*_(3,78)_ = 3.93, *p* = 0.011, η_p_^2^ = 0.13], and no differences in Animal–location task [cued recall: *F*_(3,96)_ = 2.14, *p* = 0.101, η_p_^2^ = 0.06, recognition: *F*_(3,96)_ = 0.61, *p* = 0.613, η_p_^2^ = 0.02]. Furthermore, good indices of equivalence, as assessed by intraclass correlation coefficients (ICC), were observed between task forms–Face–word AM task cued recall: ICC = 0.87, *p* < 0.001, 95%CI: 0.76–0.94, recognition: ICC = 0.86, *p* < 0.001, 95%CI: 0.74–0.93; Face–object AM task cued recall: ICC = 0.71, *p* < 0.001, 95%CI: 0.48–0.86, recognition: ICC = 0.81, *p* < 0.001, 95%CI: 0.65–0.90; Animal–location AM task: cued recall: ICC = 0.87, *p* < 0.001, 95%CI: 0.76–0.94, recognition: ICC = 0.86, *p* < 0.001, 95%CI: 0.74–0.93. Moreover, all three AM tasks showed good reliability i.e., internal consistency indices–Face–word AM task cued recall: α = 0.77–0.85, recognition: α = 0.79–0.86; Face–object AM task cued recall: α = 0.59–0.77, recognition: α = 0.74–0.85; Animal–location AM task: cued recall: α = 0.77–0.85, recognition: α = 0.76–0.80. The median correlation across tasks forms for cued recall measures was *r* = 0.56, and *r* = 0.55 for associative recognition.

To maximize the equality in terms of difficulty of the tasks, we made minimal adjustments, which were possible using a performance in the recall block as a reference point (e.g., moving a difficult-to-remember word from F4 to F1, and vice versa, or re-pairing some of the face stimuli with a different word/object within the same form to make the association more difficult), which resulted in final forms of the tasks (available on OSF, free for adaptation, and reuse [93]).

#### 2.4.10. Successfulness of Blinding Assessment

To assess the successfulness of participants’ blinding, the end-of-study guess procedure is adopted. That is, at the end of the last session (S4), the experimenter asks participants to guess in which session (S1–S4) the tES protocol they received was not real but sham stimulation. The answers are noted, and the participants are given feedback if their guess was correct or not, as a part of debriefing.

### 2.5. Data and Analytical Approach

- This experiment will generate a relatively large set of neurophysiological and behavioral data, including: Baseline cognitive measures of fluid intelligence (*Gf*), crystalized intelligence (*Gc*), visual processing (*Gv*), and processing speed (*Gs*);
- Self-report memory assessment (scores for memory satisfaction, everyday memory mistakes, and memory strategies);
- Resting state EEG (eyes-closed and eyes-open from S0, pre-tES and post-tES in S1–S4);
- Short-term AM measures during each tES condition (overall accuracy, success rate for different sequence lengths, recall times);
- Face–word AM measures following each tES condition (associative recognition–overall success rate, hit/miss scores and indexes such as d’, cued recall success rate, false recall, learning slope between two cued-recall blocks, reaction times);
- Face–object AM measures following each tES condition (associative recognition success rates and indices, cued recall success rates, reaction times);
- Animal–location AM measures following each tES condition (cued location memory success rate, assistive recognition rates and indices, reaction times);
- Task-evoked EEG data following each tES condition for three AM tasks (EEG activity during encoding, recall, recognition, repeated encoding);
- Measures of WM following each tES condition–3-back task (success rates and indices, reaction times) and Backward span (WM span and total score);
- Verbal fluency following each tES condition (number of produced words);
- Simon task-measures during each tES condition (reaction speed, error rate, congruency effect)
- pre-tES measures of depression, anxiety, stress, and overall participants’ state.

During the experiment, the data are recorded, anonymized, and stored without processing or creating any outputs. The pre-experiment data (tests of cognitive abilities) and the data from S0 (AM task and EEG for ITF) are analyzed as soon as they are collected. However, all other data (i.e., collected in S1–S4) will be analyzed upon the completion of the study. Then, the data quality checks will be performed, and potentially invalid data-segments will be flagged by the researcher blind to the experimental conditions that the data were collected under. Most of the behavioral data, including questionnaires, will be automatically scored, using the predefined scripts. When manual scoring is necessary (e.g., Face–object AM task, cued recall block), two researchers will independently score the entries, again blind to the tES condition under which the data are collected.

The overreaching strategy for the inferential statistics will be to conduct repeated measures’ ANOVA-based planned contrast. That is, each active stimulation condition will be contrasted to the sham condition under the hypothesis that tDCS, ITF-tACS, and ITF otDCS will increase AM performance relative to sham. Next, the personalized protocols will be contrasted against tDCS, to check if this type of individualization leads to more prominent effects. The primary behavioral outcomes for assessing the tES effects will be measures derived from AM tasks—specifically, associative recognition and cued recall success rates, while other cognitive measures will be treated as secondary outcomes.

In relation to neurophysiological measures, we expect to see entrainment effects of frequency-modulated tES protocols—that is, an increase in theta-frequency power following tACS and otDCS. Specifically, we will assess if resting state EEG shows pre-to-post stimulation change in power across different frequencies using a general liner model approach. The EEG data collected during AM tasks will be considered primary neurophysiological outcomes that can be analyzed alongside and in relation to behavioral measures. Again, the main dependent variables will be derived from time-frequency analysis i.e., changes in power across different frequency-bands, with primary focus on activity in theta range. In addition to that, the ERP approach will be employed to evaluate how and if different tES alter ERP components typically observed during AM tasks following sham stimulation. EEG data, both resting state and during AM tasks, will be used to offer explanation for the presence/absence of behavioral tES effects, and enhance our understanding of neurophysiological mechanisms of different tES.

Finally, all other questionnaire/behavioral measures will be treated as either control measures (e.g., verbal fluency) or moderators (e.g., sham guessing), and the most appropriate statistical model (e.g., correlational analysis, analysis of covariance, interaction effects, etc.) will be selected upon the inspection of data. Due to the novelty of the study and the size of dataset, if we come across any unexpected or incidental findings, further (exploratory) analysis will be conducted, and the findings will be treated as hypothesis-generating. For example, we might observe differential tES effects across different groups of participants (responders/non-responders, males/females, high/low performers), and/or depending on some neurophysiological or cognitive properties. However, as the study is powered for primary analysis, all additional analysis will be accompanied by sensitivity analysis [70].

## 3. Discussion

This study aims to explore the potential of using EEG recorded brain rhythms to personalize tES, thus promoting the AM functions, which is of great interest to both basic research and future clinical application. The study builds on the existing evidence that theta-activity underlines successful binding in the AM [4], and that we can enhance AM by targeting PPC as one of the tES-accessible nodes of a cortico-hippocampal network supporting this function [27,29,33,39,40,50]. However, the study extends this line of research by using the EEG data to personalize the frequency in which the stimulation would be delivered [62]. Moreover, unlike any of the previous studies, here we will compare the effects of constant anodal tDCS, ITF-tACS and ITF-otDCS. Therefore, this will be the first study to personalize tACS specifically for AM, and the first study ever to personalize otDCS. The four-tES-condition design will enable detection of tES-type specific effects and enable quantification of the potentially added value of this approach to oscillatory tES personalization.

With a few exceptions (e.g., see [94]), most of the tES studies on cognitive functions assess behavioral outcomes by using a single task to generate outcome measure(s). Here, we use multiple tasks of the same cognitive function (AM), which enables us to assess the reproducibility of potential effects. Moreover, we include several control tasks to enable making inference about the specificity/generality of the tES effects. The combination of several types of tES, as well as the assessment of online and offline behavioral outcomes alongside neurophysiological changes, will enable us to have a comprehensive overview of the potential effects, and to deepen the understanding about the mechanisms of tES for memory neuromodulation.

To overcome the heterogeneity of research designs and outcomes in tES literature, the power analysis for this study is based on previous comparable experiments conducted in our lab [39,40,50], and it takes into account the correlation between repeated measures as obtained in the pilot study for all AM tasks. Due to the novelty of research and limited evidence on the effects of different tES especially on different neurophysiological measures, the study is powered to detect at least medium effects’ sizes across all outcome measures. Despite nominally modest target sample size, the study will have sufficient power to detect effect sizes commonly reported in the literature [23,51,83]. It should be noted that, with 35–40 participants, this study will be on the higher end of the spectrum with regard to tES studies, especially for the cross-over designs [51]. As each participant will go through all four tES conditions, this study is equivalent to between-subject design with 140+ participants equally distributed across groups. Besides higher power, the main advantage of within-subject design is that each person is compared to themselves, which minimizes the effects that might stem from individual differences between participants across groups. Importantly, the study is intentionally high-powered and oversampled, as we expect some amount of data loss either due to technical (e.g., equipment malfunction, inadequate quality of the EEG signal) or statistical reasons (e.g., low internal constancy of responses, floor/ceiling effect on some measure). The quality of each measure will be assessed individually, and we will strive to exclude as little data as possible while protecting the validity of the overall dataset.

Despite all this, there are several limitations to the study design which must be highlighted. First, the sampling population is young healthy adults of probably above-average cognitive abilities. On the one hand, this adds to the feasibility of the study, as we can expect them to comply with complex experimental procedures, follow instructions, and be able to switch between the requirements of different tasks. On the other hand, it is reasonable to assume that this group of participants already functions at the optimal level, thus the effects of tES might be less pronounced than in lower performing individuals [40].

Furthermore, the participants complete several cognitively demanding tasks in a row, which might induce fatigue and lower performance towards the end of the session (S1–S4). We recognize this as a potential confounding factor; however, the duration of all tasks combined does not exceed 45-min post-tES, which is less than the usual testing time in typical student-sample studies. The potential fatigue is one of the primary reasons why a fixed order of the tasks across sessions (in contrast to counterbalancing the task order) is set. This way, each task is performed after a fixed post-tES time, and the comparison is made against the sham session with the same task order for each participant.

On the final note, it should be pointed out that this is a single-blind study—that is, at least one of the experimenters will be aware of the type of stimulation that is administered. This is mostly due to practical reasons, as in the case of the equipment/protocol malfunction, the experimenters need to be able to apply the correct person-specific protocol in each session. A double-blind design, i.e., assessment against the control condition that is masked from the participants and the study personnel is sometimes perceived as a *sine-qua-non* for adequate assessment of tES efficacy. However, experimenters can be accidently unblinded even if the stimulation device is specifically designed for double-blind design, and the studies that include EEG are at a higher risk for unblinding since an EEG during tES, or in this case in transition from tES, will show the stimulation artefacts [95]. There is additional evidence that experimenters managed to guess the stimulation condition above the chance-level even without EEG and despite all double-blind procedures set in place [96]. Therefore, we decided to set in other mechanisms to minimize potential experimenter effects, such as protocol-blind data management. In addition, we argue that using the automatic assessment tools and scoring additionally decreases the potential bias [83,97].

In summary, the study reports on the development of personalized tES protocols for targeted AM modulation and comprehensively assesses their neurophysiological and behavioral effects.

## Data Availability

All data generated in this study will be made publicly available in line with institutional and funding agency guidelines.

## Supplementary Materials

The OpenSesame code and instructions for TCP enabled EEG triggers using NIC2 can be downloaded at: https://www.mdpi.com/article/10.3390/brainsci12040472/s1.
