# Supplementary material for: Personalized Frequency Modulated Transcranial Electrical Stimulation for Associative Memory Enhancement
Source: Brain Sci. 2022 Apr 2;12(4):472. doi: 10.3390/brainsci12040472 (PMC9025454; doi:10.3390/brainsci12040472)
Supplement: Supplementary file 1 [file brainsci-12-00472-s001.zip › brainsci-1598571-supplementary.pdf]

NIC (Neuroelectrics devices e.g., Starstim) and OpenSesame

Two computers set-up is used: one for the participant to complete the task in OpenSesame software and the second one to record EEG signal with NIC2 software.

To maintain the connection and successfully send EEG triggers, 4 inline script items need to be added to OpenSesame task code.

(1) In the **prepare** section the following inline script is entered to set up TCP connection:

```
import socket

s = socket.socket(socket.AF_INET,socket.SOCK_STREAM)

HOST = '100.100.1.10' # The server's hostname or IP address

PORT = 1234 # The port used by the server

#if task is running on same computer as data capture then use 'localhost' else use IP address which is shown bottom right on capture software. The port number is 1234.

s.connect((HOST, PORT))
```

(2) To send presentation triggers (when stimuli appear on the participants' screen in sequence, triggers will mark stimulus onset) the following inline script should be added in the **run** section:

```
s.send(('<trigger>'+str(var.trigger_p)+'</trigger>').encode())

# The variable var_trigger_p is previously defined in the loop item in the OpenSesame experiment file. For example, we had two conditions (two types of stimuli), thus the values in this variable were 1 and 2.
```

(3) The inline script for triggers that appear after the keyboard response. Note: we used the IF function to send separate triggers if the participant responds correctly/incorrectly. The following code is added to the **run** section:

```
if var.correct_response ==1 : # the variable var.correct_response is where the responses log as either correct (1) or incorrect (2)

s.send(('<trigger>'+str(3)+'</trigger>').encode())

else:

s.send(('<trigger>'+str(4)+'</trigger>').encode())

#values 3 and 4 were used since 1 and 2 had been already used in a previous inline script
```

Note: We used the IF function to send separate triggers if the participant responds correctly/incorrectly.

(4) To close the TCP port. the following line at the end of experiment the **run** section should be added:

```
s.close()

# close the socket
```

When the task is running the triggers will be visible in NIC live view.
